# Total Phenolic, Flavonoid Contents, and Antioxidant Activities of Fruit, Seed, and Bark Extracts of *Zanthoxylum armatum* DC

**DOI:** 10.1155/2020/8780704

**Published:** 2020-03-16

**Authors:** Nirmala Phuyal, Pramod Kumar Jha, Pankaj Prasad Raturi, Sangeeta Rajbhandary

**Affiliations:** ^1^Central Department of Botany, Tribhuvan University, Kirtipur, Kathmandu, Nepal; ^2^Forest Research and Training Centre, Ministry of Forests and Environment, Babarmahal, Kathmandu, Nepal; ^3^Ashok Medicinal and Aromatic Plants Center, Dabur Nepal Pvt. Ltd., Janagal, Kavre, Kathmandu, Nepal

## Abstract

Natural antioxidants present in several medicinal plants are responsible for inhibiting the harmful effects of oxidative stress. These plants contain polyphenols and flavonoids that act as free radical scavengers and reduce oxidative stress and may be an alternative remedy to cure various harmful human diseases. This study aims to quantify the total phenolic and flavonoid contents (TPC and TFC) and antioxidant properties of methanol extracts of fruits, seeds, and bark of an important medicinal and aromatic plant, *Zanthoxylum armatum* collected from wild and cultivated populations in Nepal. TPC was determined by Folin–Ciocalteu colorimetric method using gallic acid as standard, and various concentrations of the extract solutions were measured at 760 nm. TFC was calculated by aluminum chloride colorimetric assay. Quercetin was used as standard, and the absorbance was measured at 510 nm. The antioxidant potential of the different extracts was estimated by DPPH free radical scavenging assay, and the absorbance was measured at 517 nm. The highest TPC value was 226.3 ± 1.14 mg GAE/g in wild fruits, and the lowest was 137.72 ± 4.21 mg GAE/g in cultivated seeds. Similarly, the highest TFC value was 135.17 ± 2.02 mg QE/g in cultivated fruits, and the lowest was 76.58 ± 4.18 mg QE/g in cultivated seeds. The extracts showed variable antioxidant properties. The fruits exhibited excellent antioxidant properties with IC_50_ values of 40.62 *μ*g/mL and 45.62 *μ*g/mL for cultivated and wild fruits, respectively. Similarly, the IC_50_ values of the bark were 63.39 *μ*g/mL and 67.82 *μ*g/mL, respectively, for cultivated and wild samples. And the least antioxidant capacity was shown by the seeds extract with IC_50_ values of 86.75 *μ*g/mL and 94.49 *μ*g/mL for wild and cultivated seeds, respectively. The IC_50_ value of the standard ascorbic acid was 36.22 *μ*g/mL. Different extracts of *Z*. *armatum* contain considerable amount of phenols and flavonoids, including antioxidant properties, suggesting the potential use of this species in pharmacy and phytotherapy as a source of natural antioxidants.

## 1. Introduction

Medicinal plants have been used in several indigenous herbal practices since very old times to cure several diseases. Herbal medication still continues to serve as an important health care system even today despite the greater advancements in modern medication systems in the recent years. Their long uses in the folk medicine and their safer implications in human health have generated much interest in them, especially in developing countries. It has now been established that medicines derived from plant products are safer than their synthetic counterparts [1].

Plant and plant-based products are the natural sources of different phytochemicals such as phenols, flavonoids, alkaloids, glycosides, lignins, and tannins. Phenols and flavonoids are the most common phytoconstituents of different fruits, vegetables, and medicinal and aromatic plants, which are responsible for antioxidant activities [2]. Due to the potential toxicological effects of synthetic antioxidants [3], natural antioxidants such as phenols and flavonoid compounds from plant origin are gaining popularity these days [4]. An antioxidant is a substance that inhibit or delays oxidative damage to the cells of the organisms by scavenging the free radicals such as peroxide or hydroperoxide and thus reducing the risk of degenerative diseases [5]. Abnormal production of free radicals may cause several severe human diseases such as cancer; Alzheimer's disease; cardiac, kidney, and liver diseases; fibrosis; atherosclerosis; arthritis; neurodegenerative disorders; and aging. Several medicinal plants have been screened for their antioxidant and other biological activities [6–8].

*Zanthoxylum armatum* DC (family: Rutaceae), commonly called as *Timur* in Nepal, is an aromatic perennial shrub or a small tree up to 6 m height with dense glabrous foliage and straight prickles. It is distributed from Kashmir to Bhutan, north-east India and Pakistan, Laos, Myanmar, Thailand, China, Japan, North and South Korea, North Vietnam, and Taiwan [9]. In Nepal, it is found at 1000 m to 2500 m from east to west [10]. It is used in traditional medicinal systems for various ailments such as cholera, diabetes, cough, diarrhea, fever, headache, microbial infections, and toothache [11–14]. The dried fruits of the plant are used as condiment and have excellent spice value. Several phytocomponents such as alkaloids, flavonoids, terpenoids, phenols, and steroids have been extracted from different parts of the plant such as fruits, seeds, leaves, and bark [13, 15, 16]. These compounds are responsible for several pharmacological activities such as antibacterial, antifungal and antihelminthic, antioxidant, anti-inflammatory, hepatoprotective, cytotoxic, larvicidal, and antispasmodic [17–21].

A lot of experiments have been carried out in *Z*. *armatum* regarding their antioxidant properties [22]. But the comparative study from different habitats and different parts of the plants is still meager, so the present study was carried out to quantify the total phenolic and flavonoid contents and evaluate the antioxidant properties in methanolic extracts of the fruits, seeds, and bark of *Z*. *armatum* collected from wild and cultivated populations. The correlation between total phenolic and flavonoid content and antioxidant activity with the habitat conditions could help to establish foundation for further studies focusing on the development of safer and inexpensive natural antioxidants for their use in therapeutic and pharmaceutical preparations.

## 2. Methods

### 2.1. Collection and Processing of Samples

The fresh fruits, seeds, and bark of *Z*. *armatum* were collected from wild and cultivated populations from Salyan district of west Nepal during May 2018. The plants were collected with the permission of Department of Plant Resources, Ministry of Forests and Environment, Government of Nepal, in accordance with article no. 10(B) of Plant Resource Research Procedure 2013 and revised 2016. This plant is not mentioned in CITES and protected plant list of Nepal. The plant was identified by Nirmala Phuyal. Herbarium of voucher specimens was prepared and deposited at National Herbarium and Plant Laboratories (KATH); NPZA 20-NPZA 50. The samples were cleaned and shade dried for a week before the extraction procedure.

### 2.2. Extraction of the Samples

The dried samples were then powdered separately in a grinder. Known weight of the powdered samples was loaded in thimble and put inside the Soxhlet apparatus. They were then successively extracted with methanol by the hot Soxhlet extraction method. The apparatus was run for 72 hours till the colored solvent appeared in the siphon for obtaining the crude extracts of the samples. After complete extraction, the solvent was evaporated in a rotary vacuum evaporator at 65°C under reduced pressure. The obtained extracts were then dried in a water bath. The dried extracts were sealed inside 20 mL sterilized culture tubes and stored in refrigerator at 2–8°C for further analysis [23].

### 2.3. Determination of Total Phenolic Content (TPC)

#### 2.3.1. Preparation of Standard Gallic Acid for Calibration Curve

Total phenolic contents (TPC) in the fruits, seeds, and bark extracts were determined by Folin–Ciocalteu colorimetric method as described by Singleton et al. [24] with some modifications. Standard gallic acid solution was prepared by dissolving 10 mg of it in 10 mL of methanol (1 mg/mL). Various concentrations of gallic acid solutions in methanol (25, 50, 75, and 100 *μ*g/mL) were prepared from the standard solution. To each concentration, 5 mL of 10% Folin–Ciocalteu reagent (FCR) and 4 mL of 7% Na_2_CO_3_ were added making a final volume of 10 mL. Thus, the obtained blue colored mixture was shaken well and incubated for 30 min at 40°C in a water bath. Then, the absorbance was measured at 760 nm against blank. The FCR reagent oxidizes phenols in plant extracts and changes into the dark blue color, which is then measured by UV-visible spectrophotometer. All the experiments were carried out in triplicates, and the average absorbance values obtained at different concentrations of gallic acid were used to plot the calibration curve.

#### 2.3.2. Preparation of Samples for Total Phenolic Content

Various concentrations of the extracts (25, 50, 75, and 100 *μ*g/mL) were prepared. The procedure as described for standard gallic acid was followed, and absorbance for each concentration of the extracts was recorded. The samples were prepared in triplicate for each analysis, and the average value of absorbance was used to plot the calibration curve to determine the level of phenolics in the extracts. Total phenolic content of the extracts was expressed as mg gallic acid equivalents (GAE) per gram of sample in dry weight (mg/g). The total phenolic contents in all the samples were calculated by the using the formula:

(1) $$\begin{array}{c} C=c\frac{V}{m}, \end{array}$$

where *C* = total phenolic content mg GAE/g dry extract, *c* = concentration of gallic acid obtained from calibration curve in mg/mL, *V* = volume of extract in mL, and *m* = mass of extract in gram.

### 2.4. Determination of Total Flavonoid Content

#### 2.4.1. Preparation of Standard Quercetin for Calibration Curve

Total flavonoid contents in the extracts were determined by aluminum chloride colorimetric assay. Stock solution (4 mg/mL) of quercetin was prepared by dissolving 4 mg of quercetin in 1 mL of methanol. This standard solution was diluted serially to make various concentrations of 0.25 mg/mL, 0.5 mg/mL, 0.75 mg/mL, and 1 mg/mL solutions. 1 mL quercetin of each concentration was added to the test tube containing 4 mL of distilled water. At the same time, 0.3 mL of 5% NaNO_2_ was added to the test tube and 0.3 mL of 10% AlCl_3_ after 5 min. Then, 2 mL of 1 M NaOH was added to the mixture after 6 min. The volume of the mixture was made 10 mL by immediately adding 4.4 mL of distilled water. The total flavonoids content was expressed as quercetin equivalents using the linear equation based on the calibration curve.

#### 2.4.2. Preparation of Samples for Total Flavonoid Content

Stock solutions of 4 mg/mL concentration in methanol of the extracts were prepared, and they were diluted serially to make different concentrations (0.25 mg/mL, 0.5 mg/mL, 0.75 mg/mL, and 1 mg/mL) solutions. Similar procedure as described for quercetin was followed for the extracts also, and the absorbance was measured by spectrophotometer at 510 nm. Readings were taken in triplicate, and the average value of absorbance was used to calculate the total flavonoid content. The flavonoid content was expressed as quercetin equivalent (mg QE/g) using the linear equation based on the standard calibration curve.

### 2.5. Antioxidant Activities

#### 2.5.1. DPPH (2,2-Diphenyl-1-picrylhydrazyl) Radical Scavenging Activity

In vitro antioxidant activities of the extracts were determined using the DPPH free radical scavenging assay described by Nithianantham et al. [25] with some modifications. This is a quick and easy method to analyze the scavenging potential of antioxidants. DPPH in oxidized form gives a deep violet color in methanol. An antioxidant compound donates the electron to DPPH, thus causing its reduction and in reduced form its color changes from deep violet to yellow. DPPH solutions show a strong absorbance at 517 nm appearing as deep violet color. Scavenging of DPPH free radical determines the free radical scavenging capacity or antioxidants potential of the test samples, which shows its effectiveness, prevention, interception, and repair mechanism against injury in a biological system.

#### 2.5.2. Preparation of DPPH Solution (0.1 M)

DPPH solution (0.1 M) was prepared by dissolving 0.39 mg of DPPH in a volumetric flask, dissolved in methanol, and the final volume was made 100 mL. Thus, prepared purple-colored DPPH free radical solution was stored at −20°C for further use.

#### 2.5.3. Preparation of Extract Solutions

Stock solution of different extracts of 1 mg/mL was prepared by dissolving required quantity of each extract in required volume of methanol. From the sample stock solution, 25, 50, 75, and 100 *μ*g/mL solutions of each extract were prepared.

### 2.6. Evaluation of Antioxidant Potential

To the sample solutions of different concentration, 1 mL DPPH solution was added and incubated at room temperature for 30 min in dark. A control was prepared by mixing 1 mL methanol and 1 mL DPPH solution. Finally, the absorbance of the solutions was measured by using a spectrophotometer at 517 nm. Ascorbic acid was used as the standard. 50% inhibitory concentrations (IC_50_ values) of the extracts were calculated from graph as concentration versus percentage inhibition. Radical scavenging activity was expressed as percentage of inhibition. IC_50_ value is the concentration of sample required to scavenge 50% of DPPH free radical. Measurements were taken in triplicate. IC_50_ of the extracts indicates the corresponding concentration in which the radical scavenging potential is 50%. The IC_50_ of the extract and standards were determined graphically.

The percentage of inhibition was calculated by using the formula:

(2) $$\begin{array}{c} \text{I}\%=\frac{\text{AC}-\text{AO}}{\text{AC}}\times 100\%, \end{array}$$

where AC = absorbance of the control (1 mL methanol + 1 mL DPPH solution), AO = absorbance of the sample solution, and I% = percentage of inhibition.

The radical scavenging activities of the extracts are expressed in terms of their IC_50_ values. The data were presented as mean values ± standard deviation (*n* = 3).

## 3. Results

### 3.1. Total Phenolic Contents (TPC)

Total phenolic contents in different extracts of fruits, seeds, and bark of *Z*. *armatum* were determined by Folin–Ciocalteu (F–C) method using gallic acid as the standard. The absorbance values obtained at different concentrations of gallic acid were used for the construction of calibration curve. Total phenolic content of the extracts was calculated from the regression equation of calibration curve (*Y* = 0.0108*x*; *R*^2^ = 0.993) and expressed as mg gallic acid equivalents (GAE) per gram of sample in dry weight (mg/g).

TPC values were higher in the fruit and bark extracts than the seed extracts. The highest TPC value was observed for the fruits followed by the bark, and the lowest was for the seeds extracts. TPC value of the cultivated fruit extract was 226.3 ± 1.14 mg GAE/g and that for wild fruit was 185.02 ± 2.15 mg GAE/g. Similarly, the value was 185.15 ± 1.22 mg GAE/g for wild bark and 171.13 mg GAE/g for cultivated bark. And for the wild seeds, the TPC value was 167.74 ± 2.63 mg GAE/g and that for cultivated was 137.72 ± 4.21 mg GAE/g (Table 1).

This is in accordance with the study of Barkatullah et al. [18], where the TPC value of ethanolic extracts of *Z*. *armatum* fruits was found to be 21.68 ± 0.44 mg/g and that of the bark was 16.48 ± 1.33 mg/g. Similarly, in another study, the TPC value of methanol extract of fruits was 366.3 mg of GAE/g [19]. The phenolic content of any plants is directly related to their antioxidant properties. Phenolic compounds act as reducing agents, hydrogen donors, and are capable of scavenging free radicals [26]. Presence of considerably good amount of phenolics in the fruits, seeds, and bark extracts of *Z*. *armatum* may contribute significantly to the antioxidant properties. Because of these properties, this plant might have been used in several traditional herbal medications.

The antioxidant response of phenolic compounds varies remarkably, depending on their chemical structure [27]. In addition, there may be some interference rising from other chemical components present in the extract, such as sugars or ascorbic acid [28]. In this study also, there were differences in the total phenolic components of the wild and cultivated fruits, seeds, and bark extracts. These differences could arise from variations in genetic backgrounds, environmental factors, agronomic practices as well [29].

### 3.2. Total Flavonoid Contents (TFC)

Total flavonoid content of the extracts was calculated from the regression equation of the calibration curve (*Y* = 0.0011*x*; *R*^2^ = 0.992) and expressed as mg quercetin equivalents (QE) per gram of sample in dry weight (mg/g). The TFC values also showed similar trends with that of TPC values. The highest TFC value was obtained for the fruits followed by the bark, and the lowest was for the seeds extracts. The highest TFC value was 135.17 ± 2.02 mg QE/g for cultivated fruit extract, and for wild fruit, it was 103.7 ± 1.39 mg QE/g. The TFC values were 111.2 ± 3.67 mg QE/g and 91.27 ± 3.13 mg QE/g for cultivated and wild barks, respectively. Similarly, the lowest TFC value was 76.58 ± 4.18 mg QE/g for cultivated seeds, and for wild seeds, it was 92.71 ± 3.14 mg QE/g (Table 2). In a previous study, the total flavonoid content of ethanolic extracts of *Z*. *armatum* fruit was 22.8 ± 1.33 mg/g and that of bark was 18.33 ± 1.22 mg/g [18], which is quite lower than that of the present study. The concentration of phenols and flavonoids also depends on the polarity of the solvents used for extraction [30].

### 3.3. Antioxidant Activity

#### 3.3.1. DPPH Assay

**Figure 1:**
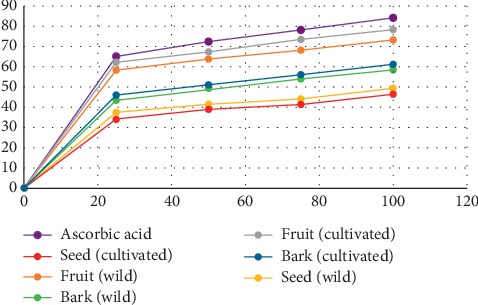
Plot of radical scavenging percentage between ascorbic acid and different samples.

Antioxidant activity of the fruits, seeds, and bark of *Z*. *armatum* was determined by DPPH free radical scavenging assay, and their reducing power was determined on the basis of their concentration providing 50% inhibition (IC_50_) values or in other words, the amount required to scavenge 50% DPPH free radicals. The mean percentage of DPPH free-radical scavenging activity at different concentrations of extracts is shown in Table 3, Figure 1. The radical scavenging activity of different extracts increased in a concentration dependent manner.

Ascorbic acid used as the standard and the different extracts showed variable antioxidant properties. The IC_50_ value of ascorbic acid was 36.22 *μ*g/mL. The higher IC_50_ value indicates lower radical scavenging activity or lower antioxidant potential. The fruits extracts had the highest antioxidant capacity compared to the seeds and bark extracts. The IC_50_ value of the fruits extracts was close to that of the standard, i.e., 40.62 *μ*g/mL and 45.62 *μ*g/mL, respectively, for the cultivated and wild fruits. Similarly, the IC_50_ value was 86.75 *μ*g/mL and 94.49 *μ*g/mL, respectively, for wild and cultivated seeds, showing least antioxidant properties. And the bark showed moderate antioxidant capacity with the IC_50_ value of 63.39 *μ*g/mL and 67.82 *μ*g/mL for cultivated and wild extracts, respectively. The antioxidant potential of fruits and bark extracts were higher in cultivated than in wild samples, whereas in seeds extracts, it was higher in wild than cultivated samples.

The antioxidant potential of different parts of *Zanthoxylum armatum* has been evaluated by various previous studies [20, 31–35]. The free radical scavenging activity of methanolic fruits extracts ranged from 59.56 to 64.85% [36], while in the present study, the scavenging percent ranged from 58.35 to 78.36% (Table 3). Similarly, the IC_50_ value of the methanolic bark extract of *Z*. *armatum* was 149.26 *μ*g/mL [34], but it was 63.39 *μ*g/mL in the present study.

The radical scavenging activity of different extracts of *Z*. *armatum* may be due to the presence of polyphenols, flavonoids, and phenolic compounds, and most of the antioxidant activity of plants is because of the phenols [37]. Natural antioxidants present in plants are responsible for inhibiting or preventing the harmful consequences of oxidative stress. DPPH assay among many other assays is one of the convenient methods for determining the antioxidant potential of plants. The presence of antioxidant substances containing hydrogen-donating groups such as flavonoids and phenols causes the methanolic DPPH solution to get reduced due to the formation of nonradical [38]. Apart from antioxidant properties, flavonoids and other phenolics also exhibit several biological activities such as antimicrobial, antiviral, and anticancer [39]. These biological and pharmacological activities are usually associated with their ability of binding proteins and free radical scavenging properties [40].

Antioxidants are tremendously important substances, which possess the ability to protect the body from damage caused by free radical-induced oxidative stress. Plant polyphenols act as reducing agents and antioxidants by the hydrogen-donating property of their hydroxyl groups [41].

## 4. Conclusions

Total phenolic, flavonoid contents, and antioxidant properties of the fruits, seeds, and bark extracts of *Z*. *armatum* were considerably good. However, these parameters were remarkably better in fruit and bark extracts as compared to the seed extracts. Some of the wild samples showed excellent results, and some of the cultivated samples showed better results. The differential TPC, TFC contents, and antioxidant properties from different habitat may plausibly be due to geographical variations in chemical constituents. The results of the present study suggested that the fruits, seeds, and bark of *Z*. *armatum* could be the potent source of natural antioxidants because of their phenolic and flavonoid contents and their remarkable scavenging effects on DPPH. So, this plant could be of greater significance in preventing several harmful human diseases. Further studies should be directed towards the extensive in vivo antioxidant activities of the plant and the relationship of individual phenolic compounds to antioxidant with different mechanisms and isolation, screening, and characterization of individual compounds responsible for antioxidant properties to authenticate their probable uses as sources of natural antioxidants as well as to validate their traditional uses in several medicinal practices.

## Figures and Tables

**Table 1:** Total phenolic contents (TPC) in different extracts of *Z*. *armatum*.

S. No.	Samples	25 (*μ*g/mL)	50 (*μ*g/mL)	75 (*μ*g/mL)	100 (*μ*g/mL)	Mean TPC value (GAE/g)
1	Fruits (wild)	181.48	185.18	186.41	187.03	185.02 ± 2.15
2	Fruits (cultivated)	225.92	227.77	224.69	226.85	226.3 ± 1.14
3	Seeds (wild)	166.66	166.66	165.43	172.22	167.74 ± 2.63
4	Seeds (cultivated)	133.33	135.18	144.44	137.96	137.72 ± 4.21
5	Bark (wild)	184.77	183.33	186.41	186.11	185.15 ± 1.22
6	Bark (cultivated)	162.93	168.51	171.6	181.48	171.13 ± 6.73

**Table 2:** Total flavonoid contents (TFC) in different extracts of *Z*. *armatum*.

S. No.	Samples	0.25 mg/mL	0.50 mg/mL	0.75 mg/mL	1 mg/mL	Mean TFC value (QE/g)
1	Fruits (wild)	101.8	105.44	103.02	104.54	103.7 ± 1.39
2	Fruits (cultivated)	138.16	134.54	132.54	135.45	135.17 ± 2.02
3	Seeds (wild)	94.52	94.54	87.26	94.54	92.71 ± 3.14
4	Seeds (cultivated)	72.72	74.54	83.62	75.45	76.58 ± 4.18
5	Bark (wild)	90.88	94.54	93.33	86.36	91.27 ± 3.13
6	Bark (cultivated)	116.36	109.08	106.66	112.72	111.2 ± 3.67

**Table 3:** Mean absorbance and IC_50_ values of extract and ascorbic acid at different concentrations.

% Inhibition (scavenging capacity)							
Concentration (*μ*g/mL)	Ascorbic acid	Fruits (wild)	Fruits (cultivated)	Seeds (wild)	Seeds (cultivated)	Bark (wild)	Bark (cultivated)
100	84.21	73.29	78.36	49.41	46.44	58.45	61.25
75	78.22	68.2	73.54	44.12	41.36	53.98	56.08
50	72.45	63.84	67.38	41.47	38.98	48.67	51.12
25	65.13	58.35	62.23	37.49	34.21	43.46	46.04
0	0	0	0	0	0	0	0
IC_50_ (*μ*g/mL)	36.22	45.62	40.62	86.75	94.49	67.82	63.39

## Data Availability

All data generated or analyzed during this study are included in this article.
